# Form-Specific Prospective Environmental Risk Assessment of Graphene-Based Materials in European Freshwater

**DOI:** 10.1021/acs.est.4c05153

**Published:** 2024-11-27

**Authors:** Hyunjoo Hong, Bernd Nowack

**Affiliations:** Empa, Swiss Federal Laboratories for Materials Science and Technologies, Technology and Society Laboratory, Lerchenfeldstrasse 5, St. Gallen 9014, Switzerland

**Keywords:** graphene based material, environmental risk assessment, MFA, SSD, PEC, PNEC

## Abstract

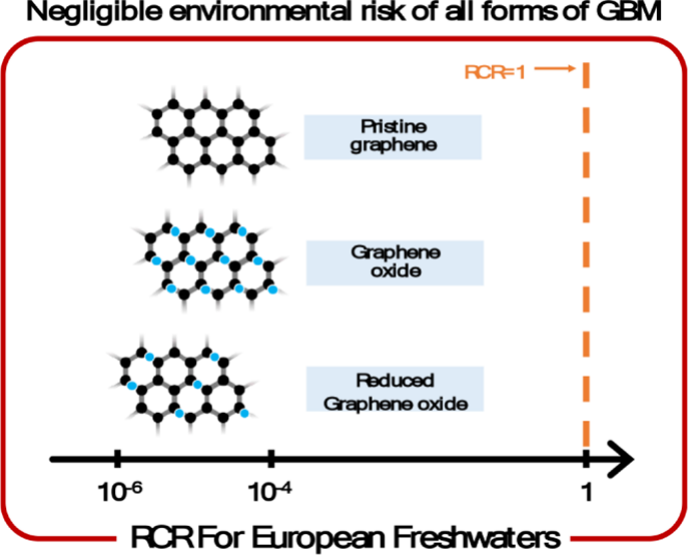

As graphene-based materials (GBMs) such as pristine graphene, graphene oxide, and reduced graphene oxide show great potential to be integrated in various applications, the need for environmental risk assessments grows, aiming to navigate the environmental fate and potential risk of the different forms of GBM. This study used dynamic probabilistic material flow analysis (DPMFA) to ascertain the prospective production volumes and distribution of GBMs within European freshwaters. The hazard assessment leveraged 113 data sets from peer-reviewed studies, addressing aquatic ecotoxicity across 26 species, by performing probabilistic species sensitivity distributions (SSD). Our findings reveal distinct environmental distribution patterns for GBM forms with predicted environmental concentrations in European freshwaters by 2030 of approximately 0.67 ng/L (SD = 0.24 ng/L) for pristine graphene and 0.33 ng/L (SD = 0.10 ng/L) for both graphene oxide and reduced graphene oxide, suggesting not only similar but notably minimal exposure levels. The risk characterization ratios (RCRs) for all forms of GBM were significantly below 1, indicating a negligible environmental risk within the scenarios assessed. Through detailed analysis considering the forms of the material, this research can inform regulatory decisions, support sustainable material design, and provide a solid foundation for a further investigation considering the environmental fate of GBM.

## Abstract 2

Dynamic probabilistic analysis predicts minimal environmental risk for graphene-based materials in European freshwaters by 2030, supporting sustainable design and regulatory decisions.

## Introduction

Graphene, renowned for its extraordinary properties such as robust strength, high conductivity, and remarkable flexibility, has attracted significant attention across a wide range of innovation fields.^1^ Its potential for revolutionizing industries from technology to materials science makes it a subject of intense research focus. The global graphene market is expected to grow significantly in the next years. A study published in the journal 2D Materials suggests that the global demand for graphene could increase to a range of 9,000 to 170,000 tons per year by 2028, with a median estimate of 30,000 tons. This indicates a wide range of possible production volumes, but reflecting a potentially huge market and wide technological advancements.^2^ Despite its potential, graphene is not devoid of limitations, which has led to the exploration of derivative materials like graphene oxide. These limitations include issues such as high production cost and integration with other materials, prompting the scientific community to seek alternatives.^3,4^

Graphene oxide, a derivative of graphene, addresses some of the limitations of graphene, thereby paving the way for novel applications ranging from electronics to biomedicine.^5,6^ This adaptation facilitates enhanced solubility and processability, broadening the scope of graphene’s applicability.^7^ Nevertheless, the transition to graphene oxide introduces its own set of challenges, particularly concerning stability and adaptability to various environments.^8^ Graphene oxide is metastable at temperatures up to 70 °C and can decompose over time into gaseous molecules and pristine graphene.^9,10^ The stability of graphene oxide is attributed to the clustering of oxygen functionalities into highly oxidized regions, which are surrounded by areas of pristine graphene.^9^ This arrangement hinders decomposition reactions. These oxygen groups can lead to structural changes, affecting the material’s stability over time.^11^ The adaptability of graphene oxide in various environments can be described by dispersity and stability. Costinas et al. highlighted that graphene oxide aqueous dispersions exhibit a meta-stability that evolves over time. The stability is initially influenced by changes in the carbon lattice and oxygen functional groups, followed by a conversion of certain oxygen groups into adsorbed water molecules.^12^ These challenges highlight the need for further refinement and optimization to fully exploit graphene oxide’s potential.

Reduced graphene oxide, another example of a graphene based material, mitigates the shortcomings of graphene oxide while retaining the advantageous properties of graphene. It is particularly useful because of its enhanced electrical conductivity and environmental resilience.^13^ It is crucial to recognize that graphene, graphene oxide, and reduced graphene oxide each exhibit distinct compositions and behaviors.^14^ Understanding these differences is essential for their effective integration into specific applications and assessing their environmental interactions.^13^ This detailed comprehension ensures that each material can be optimally utilized in a manner that leverages its strengths while mitigating potential risks.

These differences are not confined to their physical and chemical properties but extend to their environmental fate and potential toxicity, which can vary significantly among them.^15,16^ Current research underscores the importance of understanding these distinctions due to the diverse environmental risks associated with each material variant.^15^ This necessitates a comprehensive approach to evaluating their safety and impact, taking into account the full lifecycle of these materials.

Consequently, there is an emergent need for form-specific environmental risk assessment (fs-ERA) strategies that are tailored to the unique properties and behaviors of each material form. These targeted assessments are instrumental in ensuring the safe and sustainable development and utilization of graphene-based materials (GBM).^17^ Implementing these strategies allows for a nuanced understanding of each material’s environmental profile, facilitating informed decision-making in their development and application.^18,19^ By optimizing the societal benefits of these materials while minimizing their potential adverse environmental impacts, we can advance toward a future where innovation and sustainability coexist harmoniously.

Environmental risk assessment (ERA) necessitates a holistic approach encompassing both exposure and hazard assessment to comprehensively understand the potential impacts on ecosystems.^20^ Material flow analysis (MFA) is widely recognized as a well-established method for tracing the flow of substances to the environment.^21^ Integrating MFA into environmental risk assessment frameworks enables researchers to obtain detailed insights into the distribution of materials among various environmental compartments. The released amounts derived from the MFA can serve as a starting point to obtain simplified predicted environmental concentration, representing the initial release level without accounting for transformation processes and transport within the environment.^22^ In the past few years, the MFA approach has seen significant advancements, notably through the introduction of probabilistic material flow analysis (PMFA)^23^—the first methodology to fully incorporate probabilistic aspects into MFA—and dynamic probabilistic material flow analysis (DPMFA),^24−28^ which accounts for the temporal dynamics of material production volumes and their transfer coefficients. These enhancements have greatly improved the precision and relevance of material flow analyses in environmental risk assessments. Integrating the temporal dynamic and probabilistic approach may enhance the accuracy of risk assessments, and provide a solid foundation for environmental management and policy making decisions.

In a previous study using DPMFA, the complete flows of GBM through Europe’s anthroposphere into the environment were predicted, based on the complete lifecycle of all potential applications, such as electronics, drilling fluid and composites.^18^ However, the study predicted the merged flows of various GBM forms—pristine graphene, graphene oxide and reduced graphene oxide—without differentiation, estimating the release concentrations in Europe by 2030 to be 1.4 ng/L (Q5–Q95 range: 0.73–2.2 ng/L) in surface waters, 69 ng/kg (Q5–Q95 range: 48–95 ng/kg) in natural and urban soil, and 77 μg/kg (Q5–Q95 range: 54–100 μg/kg) in sludge-treated soil.^18,19^

Hazard assessment, another critical component of environmental risk asssesment, evaluates the potential adverse effects on ecological systems. The predicted no-effect concentration (PNEC), which quantitatively assesses the hazardous effects of a substance on an environmental compartment, refers to the concentration of the pollutant below which no negative impacts are anticipated in the environmental compartment of concern. One approach is employing a single toxicity end point in conjunction with an assessment factor. This process entails dividing the toxicity data point by an assessment factor, which ranges from 1 to 1,000. The European Chemicals Agency recommends different assessment factors dependent on the type of available data and the number of toxicity end points available.^29^ Generally, the fewer data points are available, the larger is the assessment factor that should be used, thereby deriving a more conservative PNEC value. This approach is regarded for its simplicity and utility in situations with limited data. However, its has potential for either overestimation or underestimation due to lack of consideration for environmental complexity and species sensitivity variation. For example, Németh et al. (2023) reported PNEC values for graphene oxide in aquatic environments to be 4–8 ng/L using the lowest effective concentration value of the test organisms from three trophic levels and applying 1,000 as an assessment factor.^30^ On the contrary, field data or mesocosm studies aim to offer insights grounded in real-world environmental dynamics, and provide a detailed understanding of substance impacts. Despite its advantages, this approach is often hampered by its resource-intensive nature and the challenge of controlling extraneous variables, such as temporal dynamics, scale and biodiversity levels.^31−33^ By estimating PNEC values based on a statistical framework, the species sensitivity distribution (SSD) is an attractive option as it can account for the variability in species sensitivity using the most sensitive data points of each species. Unlike the traditional methods, the probabilistic SSD (PSSD) methodology embraces the variability of toxicity end points for each organisms.^34^ While this method stands out for its scientific thoroughness when sufficient data is available, it is constrained by the necessity for extensive toxicity data sets. In addressing the diverse landscape of hazard assessments within the environmental risk assessment, it is essential to evaluate the hazards of different forms of GBM by selecting the most appropriate hazard assessment method for each form.

This study seeks to close the knowledge gap regarding the environmental risks posed by different GBM forms—pristine graphene, graphene oxide, and reduced graphene oxide—, offering a targeted environmental risk assessment to clearly identify their potential hazards and patterns of environmental release. By providing a more accurate depiction of GBMs’ environmental impacts, this research will enhance our ability to promote safer, more sustainable development, utilization, and policy-making for GBMs.

## Methods

### Exposure Assessment

**Figure 1:**
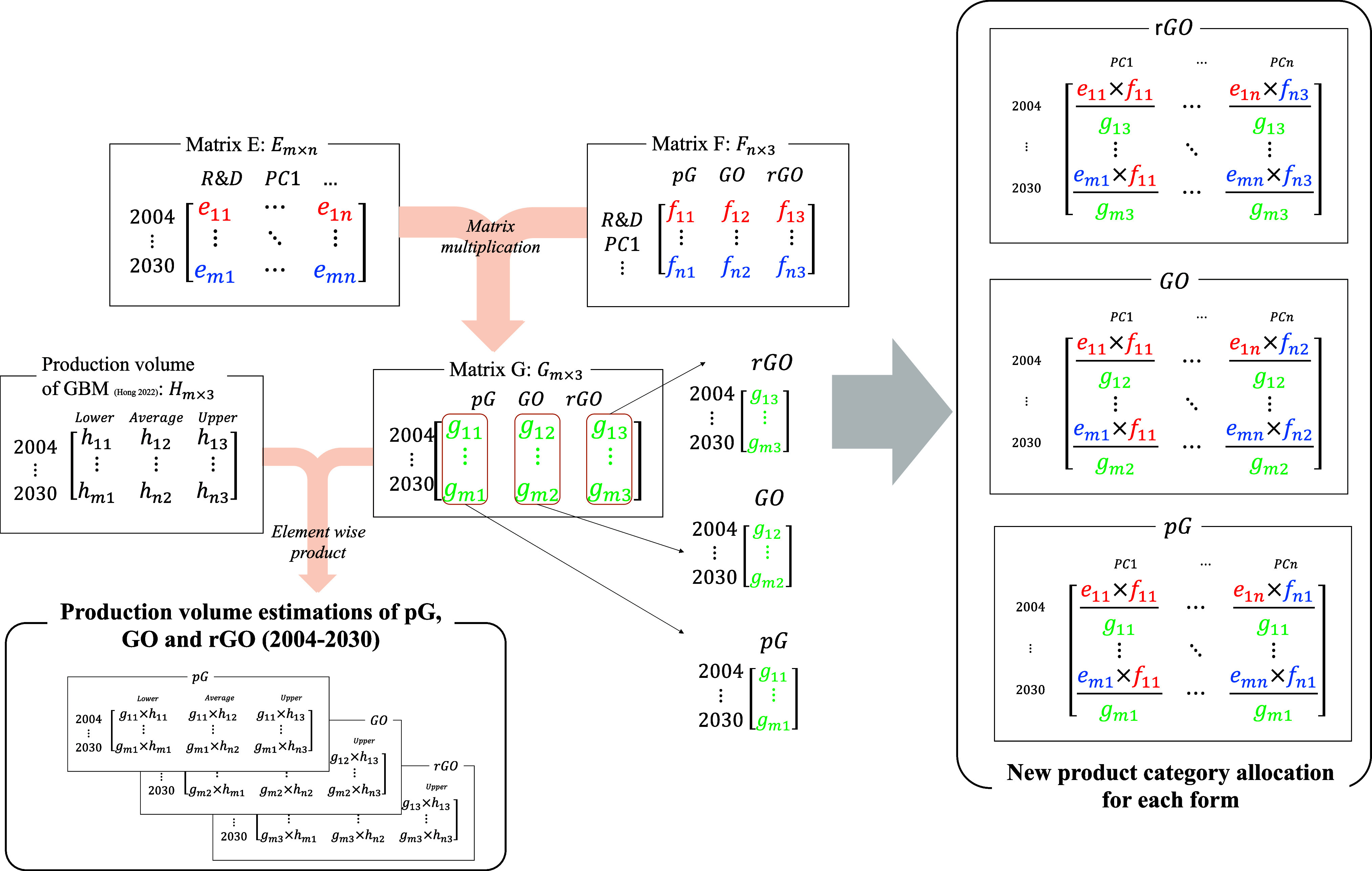
Estimation of production volume estimations and new product category allocation for each form of GBM. m is the number of years and n is the number of product categories. Matrix E is the market share in each year (sum of row is 1). Matrix F is the distribution among different forms of each product category (sum of row is 1). Matrix G is calculated by the matrix multiplication of matrix E and F. Matrix G shows the distribution among different forms in each year. For the production volume estimation of each form, matrix G was combined with the production volume estimation of total GBM from the previous study. The new product category allocation for each form is calculated using elements of matrix E, F and G.

The exposure assessment is based on the result of dynamic probabilistic material flow analysis (DPMFA).^18,19^ DPMFA requires three different input parametmers: production volume, product category allocation, and transfer coefficients. The form-specific material flow analysis of GBM updates input parameters of a previous study on the material flow analysis of GBM.^18,19^ The first input parameter that was updated for the current study is the form-specific production volume of GBM. The production volume and allocation of each form of GBM are not readily available. As the first step of estimating the form specific production volume, this study identified the allocation of the different forms of GBM within the 14 product categories that were identified in the previous study. There were two data source taken into account. Lin and colleagues (2019) reported the market fraction of pristine graphene (graphene nanoflakes (52%) and chemical vapor deposition (CVD) films (10%)) and graphene oxide (38%) (matrix A of Figure S1).^35^ They also reported the distribution of different forms of GBM across five applications (composites, conductive films and inks, energy, transistors and others) (matrix B of Figure S1). By combining these two pieces of information, we have identified the distribution across different forms of GBM within each application (matrix D of Figure 1 and Table S1). However, Lin and colleagues (2019) did not provide a separate market fraction for reduced graphene oxide.^35^ Therefore, the share of graphene oxide identified in their publication was equally distributed between graphene oxide and reduced graphene oxide. The CVD film and graphene nanoflakes are both considered to be pristine graphene in our study.

The product categories identified by Lin and colleagues (2019) did not include all product categories that the previous study on the MFA of GBM identified.^18,19,35^ A Future Markets report was another data source defining the utilized forms of GBM in each application.^36^ The utilized forms of GBM in each application and the estimated distribution among different forms in each product category that were used in further calculation are shown in Table 1. The sum of the production volume of each form of GBM in 2030 is also shown in Table 1.

**Table 1:** Utilized Forms of GBM and Estimated Distribution among Different Form Within Each Product Category^a^

			Estimated distribution among different forms in the current study (matrix F)		
Product category	Utilized forms of GBM Future Markets report^36^	Relevant product category in Lin et al. (2019)	pG	GO	rGO
aerospace and aviation^b^	CVD, GO, rGO	composite	81%	10%	10%
automotive^c^	CVD, GO, rGO	composite others	77%	12%	12%
batteries	-	energy	57%	22%	22%
sporting goods		composite	81%	10%	10%
wind turbines		composite	81%	10%	10%
construction	GO	-	0%	50%	50%
drilling fluid	GNP, GO, rGO	-	33%	33%	33%
electronics	Pristine graphene	transistors	100%	0%	0%
filtration	GO rGO		0%	50%	50%
medical	CVD, GO, rGO		33%	33%	33%
paint and coatings	CVD, GO, rGO		33%	33%	33%
textiles	CVD, GO, rGO		33%	33%	33%
tire		composite	33%	33%	33%
research and development^d^	-	-	71%	17%	12%
Production volume in 2030 (metric tonnes)			2406	526	527

a Here, pG, GO and rGO indicates pristine graphene, graphene oxide and reduced graphene oxide, respectively. All values below the decimal point were rounded.

b The distribution of form within composite.

c 90% of composites and 10% of others.

d The share for different forms in research and development field is approximated to the number of published papers (Table S2).

The estimated distribution across various GBM forms, denoted as matrix F (Table 1), was used in subsequent steps (Figure 1) to determine the form-specific production volumes of GBMs. The product category allocation used in the previous study (matrix E) multiplied by the matrix F (estimated distribution among different forms) was further combined with the production volume of GBM from the previous study (Figure 1).

The current study updates the allocation of annual product categories for each form, as depicted in Figure 1. Initially, the new ratios were computed by multiplying two key factors: the annual share of each product category (represented by matrix E), and the proportion of the form within that product category (represented by matrix F). The resulting values, denoted as *e*_*mn*_ × *f*_*nz*_ (where *m* represents the number of years, *n* the number of product categories, and *z* corresponds to the specific types of graphene—1 for pristine graphene, 2 for graphene oxide, and 3 for reduced graphene oxide), were then normalized by the values of *g*_*mz*_, adjusting the ratios for each year and form. The system boundary and the technical and environmental compartments are illustrated in Figure S2. Our model considered air as a transfer compartment through which mass flows in and out without accumulating over time. This assumption is reasonable as the time frame of the model is one year, and most airborne materials will be deposited back to the Earth’s surface within this period due to the effects of gravity, precipitation, and other removal processes.

The predicted environmental concentration (PEC) distribution provides an estimate of the average steady-state concentration of the material in surface waters, assuming a constant annual loading of the material, a fixed compartment (surface water) volume, and the residence time water to flow through the system.^37^ As a first step, the total annual amount of the substance release into European surface waters is divided by the total volume of the freshwater compartment in Europe. The volume was approximated based on the surface area and an average depth of freshwater within Europe.^38^ Further dividing the value by the residence time of water, which is assumed to be 40 days as recommended by ECHA, the calculated PEC value takes into account how long the water and the material in the waterbody remain in the freshwater compartment before flowing out.^38^ The masses of different forms of GBM in natural and urban soils and sludge-treated soils were divided by the masses of these two soil categories in Europe to derive predicted environmental concentrations.^25^

### Hazard Assessment

A search of the peer-reviewed literature was performed to collect ecotoxicological hazard data for all forms of GBM. The search was conducted using the Web of Science and Scopus databases, with keywords related to graphene-based materials (GBM) and freshwater ecotoxicity end points. After removing duplicate studies, the abstracts were reviewed to exclude any unrelated publications. The remaining studies were then carefully examined to extract the relevant ecotoxicity end points, which were compiled for the current analysis. The literature search considered papers published before August 2023 (Full list provided in Supporting Information 2: GBM Hazard data). The PSSD approach was applied for all GBM forms combined, pristine graphene, and graphene oxide as there were enough number of data points to take statistical approach. The obtained data points of the effect concentration were originally available in various dose descriptors, such as *x*% effect concentration (ECx), *x*% lethal concentration, LOEC, highest observed no-effect concentration, and NOEC. To convert acute NOEC values to chronic NOECs, they were divided by uncertainty factors (UFs) related to exposure duration (UF_t_) and dose descriptor conversion (UF_dd_) as outlined by Wigger at al (2020).^34^ This PSSD process involves creating species-specific NOEC probability distributions, then running a Monte Carlo simulation with 10,000 iterations to derive PNEC values, ultimately determining the hazardous concentration for 5% of species (HC_5_) as the PNEC for freshwaters. Due to lack of data, the PNEC estimation of reduced graphene oxide was calculated by dividing the most sensitive end point among fish, algae and daphnids by the assessment factor of 1,000, according to the ECHA guidance.^29^

### Environmental Risk Assessment

The risk of ENM was characterized by the risk characterization ratio (RCR) distribution. The RCR distribution were derived by dividing the PEC distribution by the PNEC distribution for GBM, pristine graphene and graphene oxide. The RCR distribution of reduced graphene oxide was derived by dividing the PEC distribution by the single PNEC value estimated by deterministic approach.

## Results

### Mass Flow Diagrams

**Figure 2:**
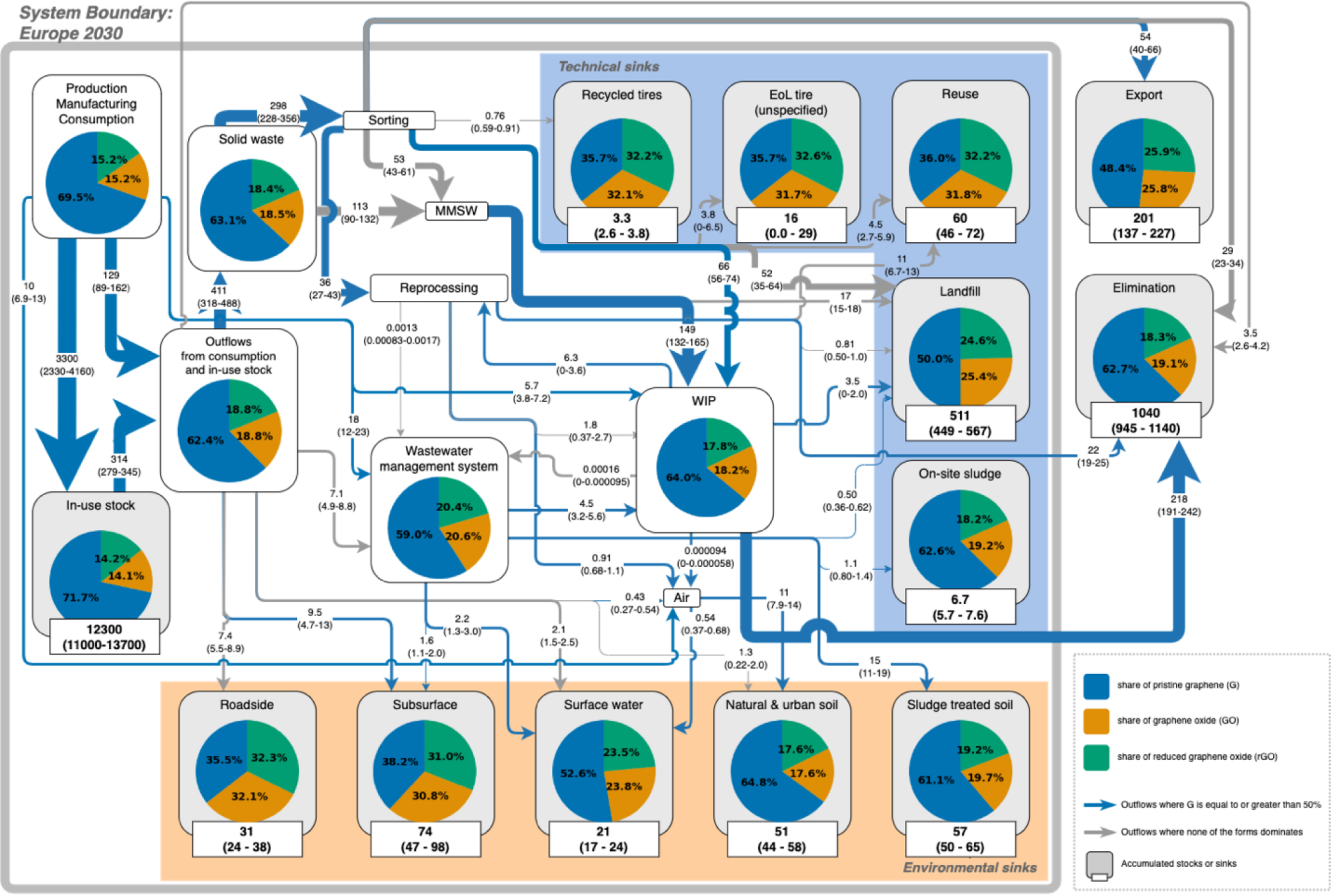
Projected European 2030 GBM flow diagram (in metric tonnes). The arrow thickness reflects the mean flow. The mean and the range between 25th percentile and 75th percentile are stated on each arrow. The mean for sink shows accumulated outlows into each sink over the years. Means, 25th and 75th percentiles smaller than 100 are rounded to two significant figures; Means, 25th and 75th percentiles greater than or equal to 100 are rounded to three significant figures. MMSW: mixed municipal solid waste; WIP: waste incineration plant; WWTP: wastewater treatment plant.

Figure 2 shows the mass flows, stocks, and sinks of pristine graphene, graphene oxide, and reduced graphene oxide for the year 2030. The pie charts in Figure 2 illustrate the average distribution of the different forms of GBM in each compartment. Most of the compartments have a similar distribution patterns as the production compartment, which is the first compartment shown at the top left in the flow diagram (59–66% of pristine graphene and the rest equally distributed between graphene oxide and reduced graphene oxide).

Elimination, accounting for 1040 t of the mass of GBM destroyed after incineration and leaving the system, shows a similar pattern of the share among different forms as the production compartment (Figure S3a). Landfill, the largest final sink within the system, contains a lower proportion of pristine graphene (50%) because it receives inflow mainly from drilling waste after sorting. The difference between proportions of pristine graphene and other forms is shown in Figure S3b. The distribution of various forms GBM in the subsurface differs significantly from that observed in production compartment. The primary pathway for GBM to enter the subsurface is through direct discharge from its application in drilling fluids. Given that all forms of GBM are utilized in drilling fluids and the specific market size for each form remains unknown, it was assumed that the market share for drilling fluid is evenly divided among the different forms. Consequently, it was estimated that the various forms of GBM are shared equally within the subsurface (Figure S3c).

### Predicted Environmental Concentrations in Europe

**Figure 3:**
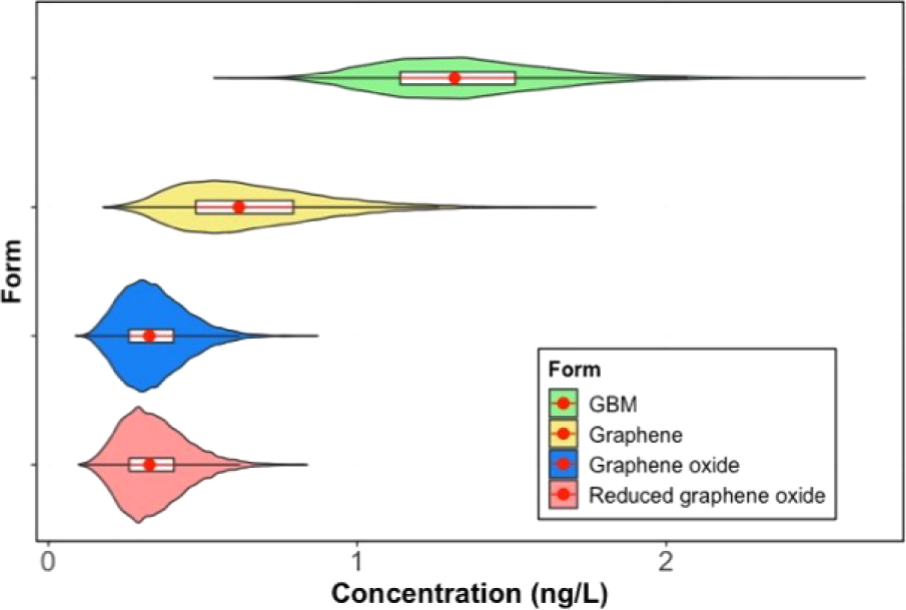
Predicted environmental concentration of GBM and all forms of GBM in European freshwaters in 2030. The red dots show the means and the red lines show the interquartile range of the distributions.

The distribution of predicted environmental concentration (PEC) was calculated based on the volume of the environmental compartments (Figure 3). As the previous study reported, the predicted average release concentration of GBM was estimated to be 1.4 ng/L in surface waters, 69 ng/kg in natural and urban soil, and 77 μg/kg in sludge-treated soil.^18,19^ The current study differentiated the various forms of GBM and predicted their respective PECs in surface waters as 0.71 ng/L (Q5–Q95 range: 0.37–1.18 ng/L) for pristine graphene, 0.34 ng/L (Q5–Q95 range: 0.19–0.54 ng/L) for graphene oxide, and 0.34 ng/L (Q5–Q95 range: 0.19–0.54 ng/L) for reduced graphene oxide.^11^ The concentrations of these forms were found to be comparable, indicating a similar level of exposure in surface water. PECs for natural and urban soils were estimated at 44.6 ng/kg (Q5–Q95 range: 31.1–60.1 ng/kg) for pristine graphene and 12.0 ng/kg (Q5–Q95 range: 7.9–17.6 ng/kg) for graphene oxide and 12.1 ng/kg (Q5–Q95 range: 8.0–17.6 ng/kg) for reduced graphene oxide, as shown in Table S4 and Figure S4. In sludge-treated soil, PECs were determined to be 46.2 μg/kg (Q5–Q95 range: 32.0–67.6 μg/kg) for pristine graphene and 15.4 μg/kg (Q5–Q95 range: 11.1–20.4 μg/kg) for graphene oxide and 15.3 μg/kg (Q5–Q95 range: 11.0–20.1 μg/kg) for reduced graphene oxide, also noted in Figure S4 and Table S4.

### Hazard Assessment

As presented in the Table S3, our study incorporated 113 data points to construct the PSSDs for GBM and three different forms of GBM. Of these, graphene oxide accounted for the largest proportion of data points (72%), followed by pristine graphene at 23% and reduced graphene oxide at 4%. Except for reduced graphene oxide, the data points for each form were statistically sufficient to build PSSDs. In the case of reduced graphene oxide, the challenges were 2-fold: not only was the quantity of data points limited, but also the diversity of species covered was inadequate to represent the entire aquatic ecosystem. For example, there were no ecotoxicity studies of reduced graphene oxide involving fish nor daphnids. Consequently, for the risk assessment of reduced graphene oxide, the PNEC_AF_ was calculated based on the most sensitive species and used to estimate the risk characterization ratio. Therefore, PNEC_AF_ was calculated by dividing the lowest toxicity data point, 34 mg/L (*C. pyrenoidosa*) by an assessment factor of 1,000.

**Figure 4:**
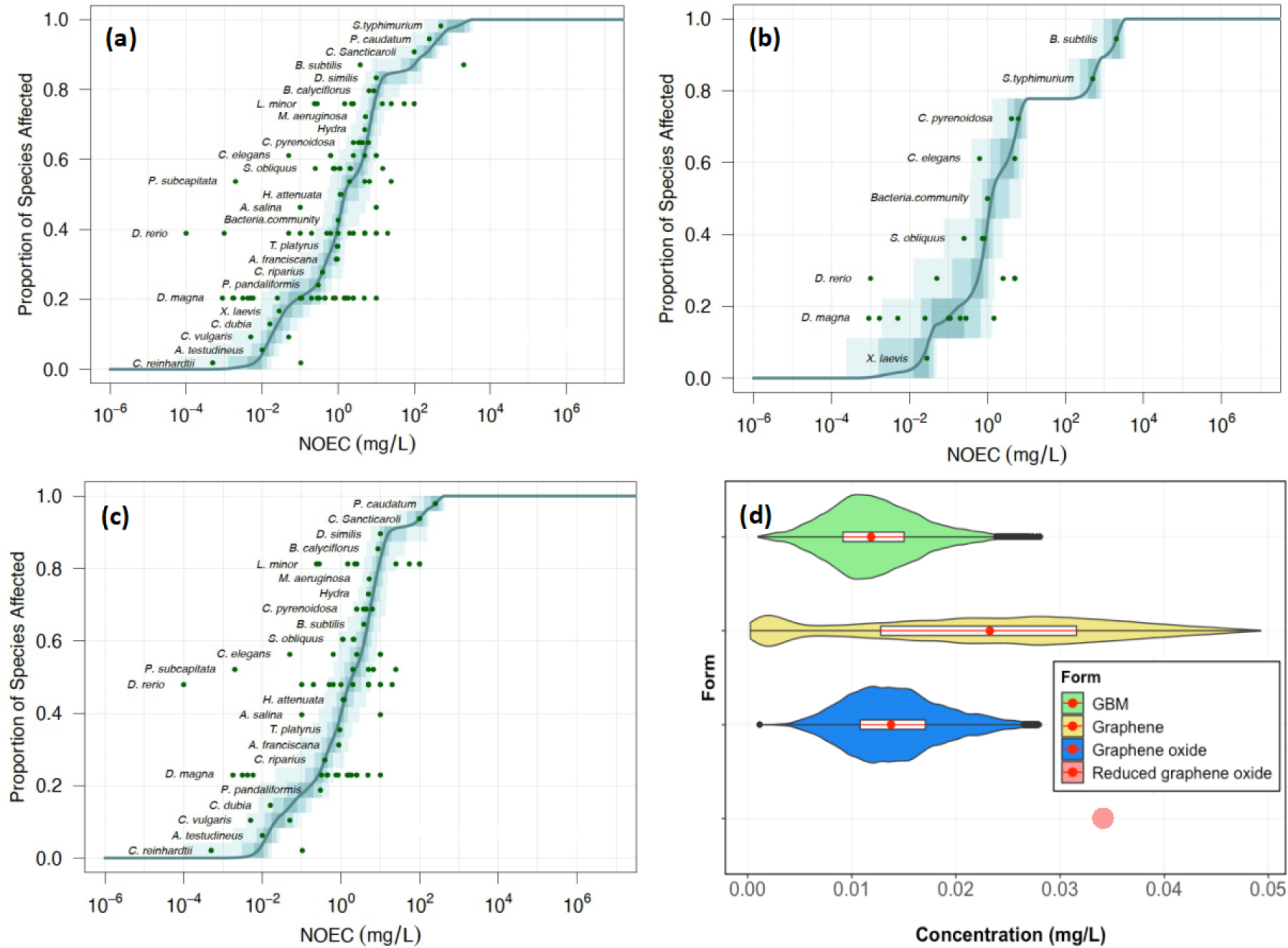
Probabilistic species sensitivity distribution analysis of GBM (a), pristine graphene (b), graphene oxide (c), and PNEC estimation of GBM and all forms of GBM (d). The values are shown in Table S5.

The form-specific PSSDs of GBM and of all forms of GBM are shown in Figure 4. The PSSD of pristine graphene was built on the basis of 26 points of 9 species. *X. laevis* was found to be the most sensitive species, with 28 μg/L, which is slightly above the HC_5_ of pristine graphene. For graphene oxide, the most sensitive species with a mean of the NOEC values below the HC_5_ was *C. reinhardtii* (3.0 μg/L).

The PNEC values (equal to HC_5_) extracted from the PSSDs and the deterministic value for reduced graphene oxide are summarized in Figure 4d. The PNEC values calculated by the assessment factor, specified by “AF” are given on the column of the mean value in Table S5. The PNECs of all different forms of GBM have the same order of magnitude. The least toxic form was the reduced graphene oxide (mean of PNEC, 34 μg/L).

### Environmental Risk Assessment

**Figure 5:**
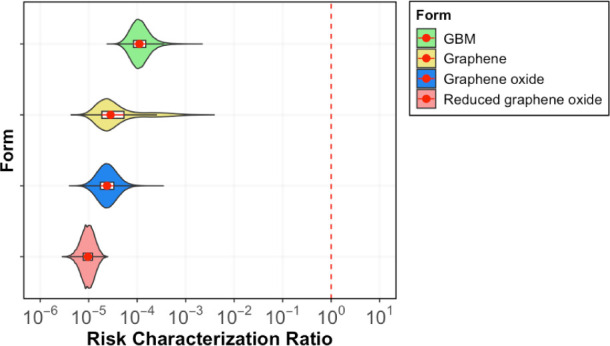
Violin plot of the risk characterization ratio (RCR) of GBM and different forms of GBM. The right side of the dashed line (red) represents the area of potential environmental risks. The red vertical line indicates the RCR of 1. The red dots show the means and the red lines show the interquartile range of the distributions.

The risk characterization ratio of GBM and of all forms were calculated using the PEC and PNEC values. These results are illustrated in Figure 5. The risk characterization ratio values for GBM and its various forms were found to be much lower than the regulatory trigger value of 1, with the mean values of these distributions ranging between 10^–4^ and 10^–6^. Specifically, GBM exhibited the highest risk characterization ratio. This elevated ratio is attributed to its lowest PNEC compared to other forms and a high PEC, which represents the aggregate of all GBM variants. Nevertheless, the mean of the GBM distribution is 4 orders of magnitude lower than 1. Reduced graphene oxide exhibited the lowest mean risk characterization ratio, being 5 orders of magnitude lower than 1. All estimations of the risk characterization ratio exhibited comparability across the different forms of GBM.

## Discussion

A material flow analysis of GBM combining all forms of GBM was already available previously.^18,19^ However, GBM is a diverse group of materials with different properties and thus a risk assessment should consider these different forms. In the current study, we have therefore disaggregated the flows of GBM into the different forms by determining form-specific transfer coefficients. Transfer coefficients represent one of the three important pieces of input data essential for conducting material flow analyses. The current study has quantified the distribution of GBMs—specifically pristine graphene, graphene oxide, and reduced graphene oxide—across various product categories. This refined approach allows for the identification of the predominant form within each flow, enabling the determination of the distribution of the different forms for each compartment and sink.

The distribution among the different forms of GBM across various product categories was derived from the available literature^35,36^ and is based on the specific forms of GBM utilized in each application. In cases where detailed information on the forms of GBM used was unavailable, we defaulted to an assumption of uniform distribution across the different GBM forms within an application. This assumption of uniform distribution was applied to five out of 14 product categories, namely medical applications, drilling fluids, paints/coatings, tires and textiles. The MFA results indicated that the distribution patterns at the production stage predominantly determine the flow of materials but the distribution in some sinks is closely linked with specific product categores. For instance, three specific sinks—roadside soils, recycled tires, and the EoL stage of tires—demonstrate a uniform distribution of GBM forms, with the automotive sector being the sole source of the inflow. Subsurface is another sink that receives inflow only from one product category, the drilling fluid. The provisional assumption of equal distribution between the three different forms of GBM offers a basis for future refinements and can be updated in subsequent studies when more information becomes available.

Our findings reveal that the majority of the compartments and sinks exhibit a distribution pattern of the different GBM forms similar to that of the production compartment, which serves as the origin of the material flows. For instance, elimination, the biggest sink of the system and the waste incineration plants, to which most of the mass flow after solid waste is directed, reflects the distribution found in the production compartment. Nevertheless, certain sinks, such as recycled tires and landfills, exhibit a different distribution among the three forms. This divergence can be attributed to these sinks receiving major inflows from specific uses that differ in the distribution of the forms from those associated with the production compartment. Specifically, sinks related to automotive applications—recycled tires, EoL of tires, and roadsides—demonstrate an equal distribution among different forms. This equal distribution is based on the assumption that the various forms used in automotive applications are equally distributed, highlighting the influence of specific use patterns of the distribution profiles within different sinks. In the case of drilling fluid, we similarly assume a uniform distribution among its diverse forms within the product category. The graphene report by Future Markets has identified all three forms as being employed in drilling fluids.^36^ Pristine graphene, while it is facing challenges related to cost, difficulties in large-scale production, and poor dispersity, is not the predominant form used in drilling fluids.^39^ However, the production costs for graphene, a significant factor for high-volume applications, have been decreasing over the last past decades. Without additional data on the distribution among different forms, assuming a uniform distribution appeared to be the most logical approach. Although the most conservative approach might be to consider the use of the most hazardous form in drilling fluid, it is less realistic to assume that a single form dominates the use. Our current assumption provides a solid basis for a scenario that can be further refined. This assumption, however, results in a decreased proportion of pristine graphene in landfills compared to the production phase. Landfills receive a significant influx of drilling waste during the sorting phase, where the distribution among different forms notably diverges from that observed during production. Furthermore, the subsurface is another sink that was significantly affected by drilling fluid. With the distributions of GBM across various forms in drilling fluids assumed to be uniform, the subsurface which is primarily influenced by the influx from the drilling fluid, was also predicted to be uniform.

This detailed segmentation enhances our comprehension of the distribution dynamics of GBM forms throughout their lifecycle, providing insights into their prevalence and flow patterns across various stages. Enhancing the granularity of information regarding the distribution of GBM forms within each product category could significantly improve the precision of existing material flow analyses. However, a general conclusion from the model is that we do not expect large variations in the distribution between different compartments. There is not one compartment identified that is dominated by a specific form. The majority of produced materials are treated as solid waste, resulting in a similar distribution across all subsequent compartments. This is in contrast to other nanomaterials for which different forms have been assesse, e.g., nanosilver, which is primarily released during the use phase and exhibits highly reactive properites.^40^ This is caused by the large variety of different uses of GBM and that different forms are suited for many applications.

In a preceding study, the PEC of GBM was estimated at 1.4 ng/L, encompassing the masses of pristine graphene, graphene oxide, and reduced graphene oxide.^18^ Our current research further refines this estimate by disaggregating the total mass of GBMs into their respective forms and calculating form-specific PEC values. We derived individual PEC values of 0.71 ng/L for pristine graphene, 0.34 ng/L for graphene oxide and 0.34 ng/L for reduced graphene oxide, resulting in a combined sum of 1.4 ng/L in the updated model. The relatively low PEC estimations indicates that none of the forms of GBM constitutes an environmentally relevant pollutant. Nonetheless, it is imperative to recognize the considerable uncertainties surrounding the current and future production volumes of GBM, as well as their distribution across different forms.

In the exposure assessment, we assumed an equal distribution between graphene oxide and reduced graphene oxide. Currently, there is limited information available regarding the specific shares for different forms in each application. Production costs could serve as a useful indicator for predicting which form might become more prevalent. The production of reduced graphene oxide involves additional processing steps compared to graphene oxide, potentially leading to higher costs. Nonetheless, the reduction of graphene oxide is an attractive method for achieving graphene-like properties. Consequently, it remains challenging to predict which form will ultimately dominate. Thererfore, assuming an equal distribution between graphene and reduced graphene oxide effectively captures the stochastic nature of the market in the absence of comprehensive data. Ultimately, varying the assumed distribution between the two forms may influence the RCR, but it is expected to remain significantly below 1.

This study employed a boundary scenario approach, laying the groundwork for discussion on potential worst-case environmental scenarios. Moreover, to reinforce the robustness of the predictive model and to provide a more comprehensive assessment of GBMs’ environmental impact, it is advisable to integrate the results of material flow analysis with environmental fate models, such as SimpleBox4Nano^41^ or NanoFate^42^ to calculate true PEC values considering also environmental fate processes. This integration is crucial for a detailed understanding of GBMs’ ecological impacts, however, the inclusion of fate processes will result in lower PEC values in the freshwater compartment as agglomeration and sedimentation will decrease the suspended concentration. Also, although the current study provides regional PEC values for GBM and its various forms, local releases can lead to significantly higher concentrations in specific areas. For example, a TiO_2_ spill from a truck accident in France in 2011 resulted in elevated local concentrations.^43^ Such accidental release can temporarily increase the PEC in a localized area, which may cause higher RCR values. Thus, refining the RCR by considering the environmental fate of each form of GBM remains critical, as it allows for a more precise assessment of potential risks in both regional and local contexts. Furthermore, while the current study concludes that there is no predominant form of GBM across different compartments on a regional scale, local variations in environmental conditions and release scenarios may lead to specific forms being more prevalent in certain areas. Integrating material flow analysis with environmental fate models enables a more comprehensive understanding of these local differences and their potential ecological implications.

In addition to surface water, we also provide form-specific PEC values for natural and urban soils, as well as sludge-treated soil. While the determination of the hazard and risk analysis for soil systems was not in the focus of this initial investigation, it can be derived by integrating our PEC findings with a further detailed hazard assessment of different forms of GBM for the soil ecosystem.

The PNEC values for various forms of GBM were calculated to enable the form-specific environmental risk assessment. The PNEC for pristine graphene and graphene oxide was determined using the PSSD approach. On the other hand, the data available for estimating the PNEC of reduced graphene oxide were too scarce to build a SSD,^29^ with only five data points, each corresponding to a single species. This scarcity extended not only to the total number of data points but also to the diversity of species represented, encompassing algae, plankton, aquatic freshwater plants, and crustaceans. The range of organisms failed to represent the broader ecosystem, notably lacking studies on fish. Additionally, when comparing the sensitivity of organisms across all forms of GBM, those most affected by GBM were not included in the tests with reduced graphene oxide. Consequently, for reduced graphene oxide, a deterministic approach was adopted. This involved dividing the lowest observed adverse effect level for the most sensitive species (*C. pyrenoidosa*: 34 mg/L) by an assessment factor of 1,000, representing a conservative strategy to ensure environmental safety.^29^ This reliance on a assessment factor approach for estimating the PNEC of reduced graphene oxide, due to the limited and nonrepresentative data, underscores a significant gap in our understanding of its environmental risks. This approach, which utilizes the most sensitive value from studies on *C. pyrenoidosa* with a conservative assessment factor, is a cautious measure but also highlights the need for a more ecologically relevant data set. The absence of data on key aquatic species like fish, and the narrow focus on a limited range of organisms, reveals a critical knowledge gap that compromises the accuracy of risk assessments and inhibits informed comparisons of the environmental risks posed by different forms of GBM. However, as we have used a very conservative approach to determine the PNEC and the final risk assessment revealed only a very low risk also for reduced graphene oxide, improving the database could also result in an increased PNEC for reduced graphene oxide as smaller assesment factors are used within the SSD approach.

To address these deficiencies and improve the environmental risk assessment of GBMs, there is a need for additional ecotoxicity studies encompassing a wider spectrum of species, including those not previously considered. Expanding research to cover important taxa such as fish and sensitive organisms like *C. dubia* will enable a more accurate, representative, and comprehensive evaluation of the potential environmental impacts of GBM. Such efforts will enhance the comparability of PNEC values across different GBM forms, contributing to more effective environmental protection and management strategies.

The RCR for all the different forms of GBM were found to be significantly lower than 1, indicating a low risk according to our study’s prospective and probabilistic approach. Despite the potential for a substantial increase in demand for these materials, our analysis suggests that their environmental impact remains minimal, attributed to the combination of low environmental exposure concentrations and high PNECs. However, it is essential to refine the RCR for each form of GBM by considering their fate in the environment. This notion is supported by the findings of Shams and colleagues (2019), who demonstrated variations in the oxidation and photodegradation rates among different forms of GBM, with reduced graphene oxide exhibiting the slowest degradation.^44^ Consequently, the environmental fate of each GBM form may differ, resulting in PEC values distinct from those estimated at the point of release, as determined in our current study. These insights underscore the importance of continually reassessing and refining the risk assessment methodologies to accurately evaluate the environmental impact of GBMs across their full lifecycle.

The current study successfully characterized the form-specific risk of GBM in European freshwaters, providing valuable insights into the environmental implications of these materials. Our findings not only contribute to understanding the potential risks associated with GBM but also highlight the importance of considering the different fates of each GBM form in environmental risk assessments. By delineating the unique environmental behavior and impacts of pristine graphene, graphene oxide and reduced graphene oxide, our research enhances the precision and comprehensiveness of material flow analyses and risk assessments. This detailed understanding of GBM’s environmental dynamics can inform regulatory decisions, guide sustainable material design, and support effective management strategies to minimize environmental impacts.

## Data Availability

The codes, input files, and raw results are available for download at Zenodo: https://doi.org/10.5281/zenodo.13374929.

## Supporting Information Available

Additional diagrams and tables of methods and results; steps of matrix multiplication for the material flow assessments (Figure S1); distribution of different forms of GBM within each application (Table S1); proportional utilization of different graphene-based materials (GBM) in research and development (Table S2); system boundary for the material flow analysis (Figure S2); overview of data used in the hazard assessment (Table S3); example of probability distributions of GBM masses in different sinks (Figure S3); predicted environmental concentration of GBM and all forms of GBM in 2030 in European sludge treated soil and natural and urban soil (Figure S4); summary of the predicted environmental concentration distribution of pristine graphene, graphene oxide and reduced graphene oxide in 2030 (Table S4); summary of the predicted no effect concentration (PNEC) distributions for the different forms of GBM (Table S5) (PDF)
GBM hazard data (XLSX)
