# Supplementary material for: Form-Specific Prospective Environmental Risk Assessment of Graphene-Based Materials in European Freshwater
Source: Environ Sci Technol. 2024 Nov 27;58(49):21750–9. doi: 10.1021/acs.est.4c05153 (PMC11636196; doi:10.1021/acs.est.4c05153)
Supplement: Supplementary file 1 — es4c05153_si_001.pdf [file es4c05153_si_001.pdf]

# Form-specific prospective environmental risk assessment of graphene-based materials in European freshwaters

Hyunjoo Hong[1], Bernd Nowack[1]*

[1] Empa, Swiss Federal Laboratories for Materials Science and Technologies, Technology and Society Laboratory, Lerchenfeldstrasse 5, 9014 St. Gallen, Switzerland

*corresponding author: bernd.nowack@empa.ch

# Supporting Information 1

Number of pages in supporting information: 5

Number of figures: 4

Number of tables: 5

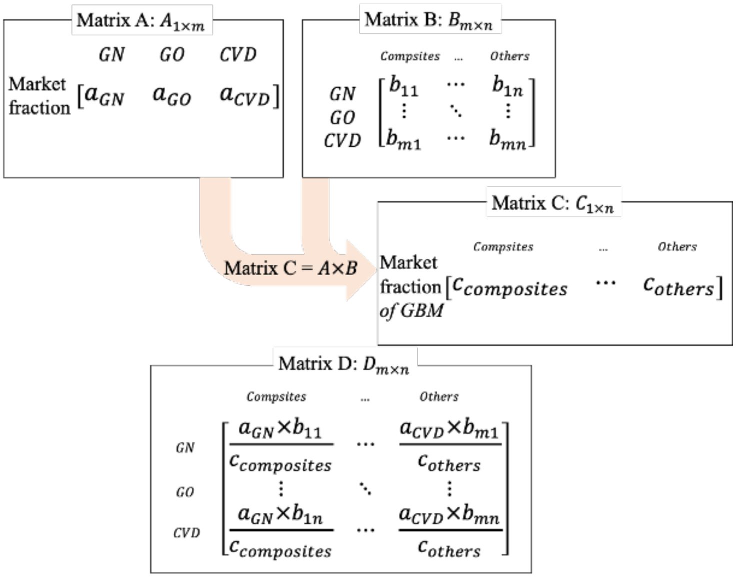


Figure S 1: Matrix A shows the market share of different GBM forms (sum of each row equals 1). Matrix B depicts the application distribution for each GBM form (sum of each row equals 1). Matrix C represents the market share of GBMs across various applications. Matrix D details the distribution of GBM forms within each application (sum of each column equals 1). Here, m=3 indicates the number of GBM forms, and n=5 represents the number of applications in Lin et al. (2019).[23] GN: graphene nanoflakes; GO: graphene oxide; CVD: chemical vapor deposition films.

Table S 1: Distribution of different forms of GBM within each application (Matrix D) calculated based on the estimation of Lin and colleagues (2019) [23]. Here, CVD, GO and rGO indicates chemical vapor deposition, graphene oxide and reduced graphene oxide, respectively. The sum of each column is 100%. However, all values below the decimal point were rounded for presentation and slightly modified to illustrate that sum of each column is may not sum to 100%.

| | **Composite** | **Conductive films and inks** | **Energy** | **Transistors** | **Other** |
|---|---|---|---|---|---|
| **Pristine Graphene** (graphene nanoflake + CVD film) | 80% | 63% | 56% | 100% | 37% |
| **GO** | 10% | 18% | 22% | 0% | 31% |
| **rGO** | 10% | 18% | 22% | 0% | 31% |

Table S 2: Proportional Utilization of Different Graphene-Based Materials (GBM) in Research and Development. This table presents an analysis of the various forms of graphene-based materials and their respective shares in research and development activities. It quantitatively details the utilization frequency and scope of each GBM type, underscoring their importance and unique applications in scientific and technological advancements.

| | | | |
|---|---|---|---|
| WoS Search keywords | TI=("graphene" NOT "graphene oxide" NOT "reduced graphene oxide") OR AB=("graphene" NOT "graphene oxide" NOT "reduced graphene oxide") | TI=("graphene oxide" NOT "reduced graphene oxide") OR AB=("graphene oxide" NOT "reduced graphene oxide") | TI=("reduced graphene oxide") OR AB=("reduced graphene oxide") |
| hit | 154609 | 44366 | 30343 |
| ratio | 0.67421223 | 0.19346933 | 0.13231844 |
| Scopus | intitle:"graphene" -intitle:"graphene oxide" -intitle:"reduced graphene oxide" intext:"graphene" -intext:"graphene oxide" -intext:"reduced graphene oxide" | intitle:"graphene oxide" -intitle:"reduced graphene oxide" intext:"graphene oxide" -intext:"reduced graphene oxide" | intitle:"reduced graphene oxide" intext:"reduced graphene oxide" |
| hit | 171000 | 31500 | 25800 |
| ratio | 0.74568939 | 0.13736384 | 0.11250752 |
| average | 71% | 16.5% | 12.5% |

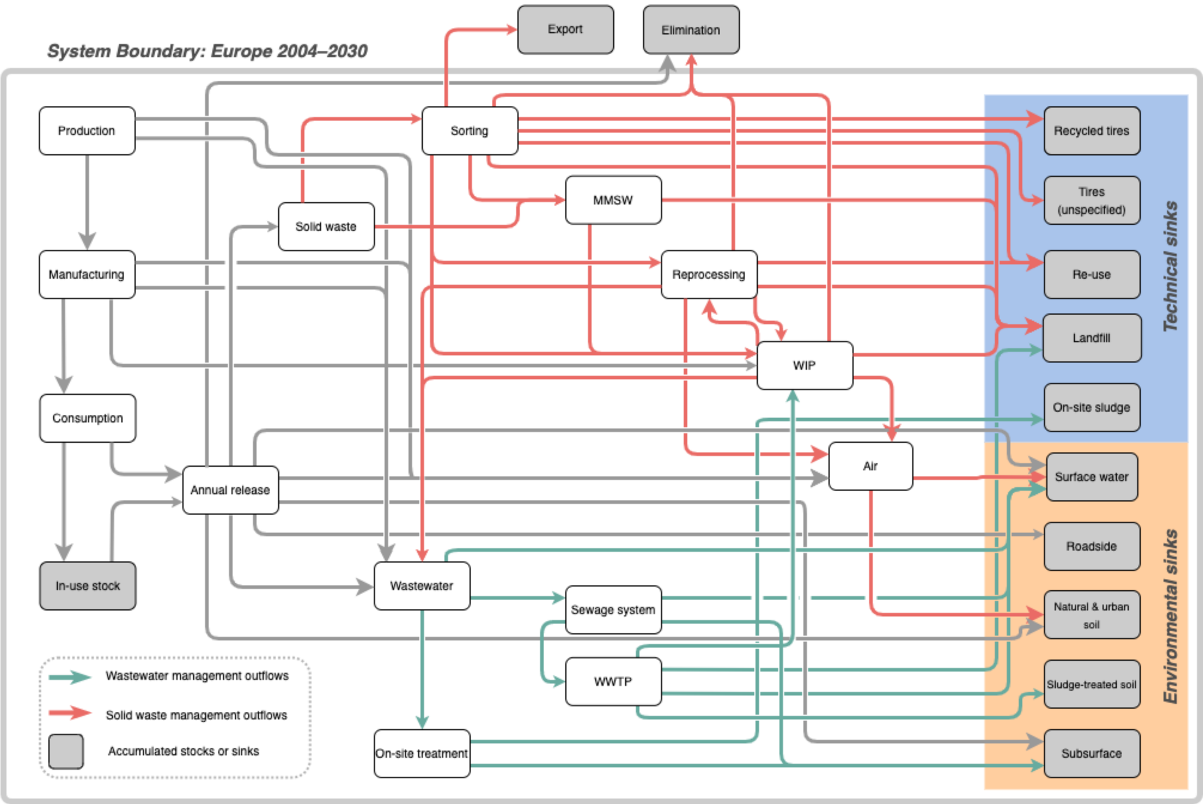


*Figure S2. System boundary for the material flow analysis. Technical compartments and processes are shown in white boxes. Gray boxes represent sinks and flows leaving the system boundary. MMSW: Mixed municipal solid waste; WIP: Waste incineration plant; WWTP: Wastewater treatment plant.*

Table S 3: Overview of data used in the hazard assessment. GBM: graphene based material.

| | GBM | Pristine graphene | Graphene oxide | Reduced graphene oxide |
|---|---|---|---|---|
| No. of data points | 113 | 26 | 81 | 5 |
| No. of species | 26 | 9 | 24 | 5 |
| No. of chronic data points | 46 | 8 | 34 | 4 |

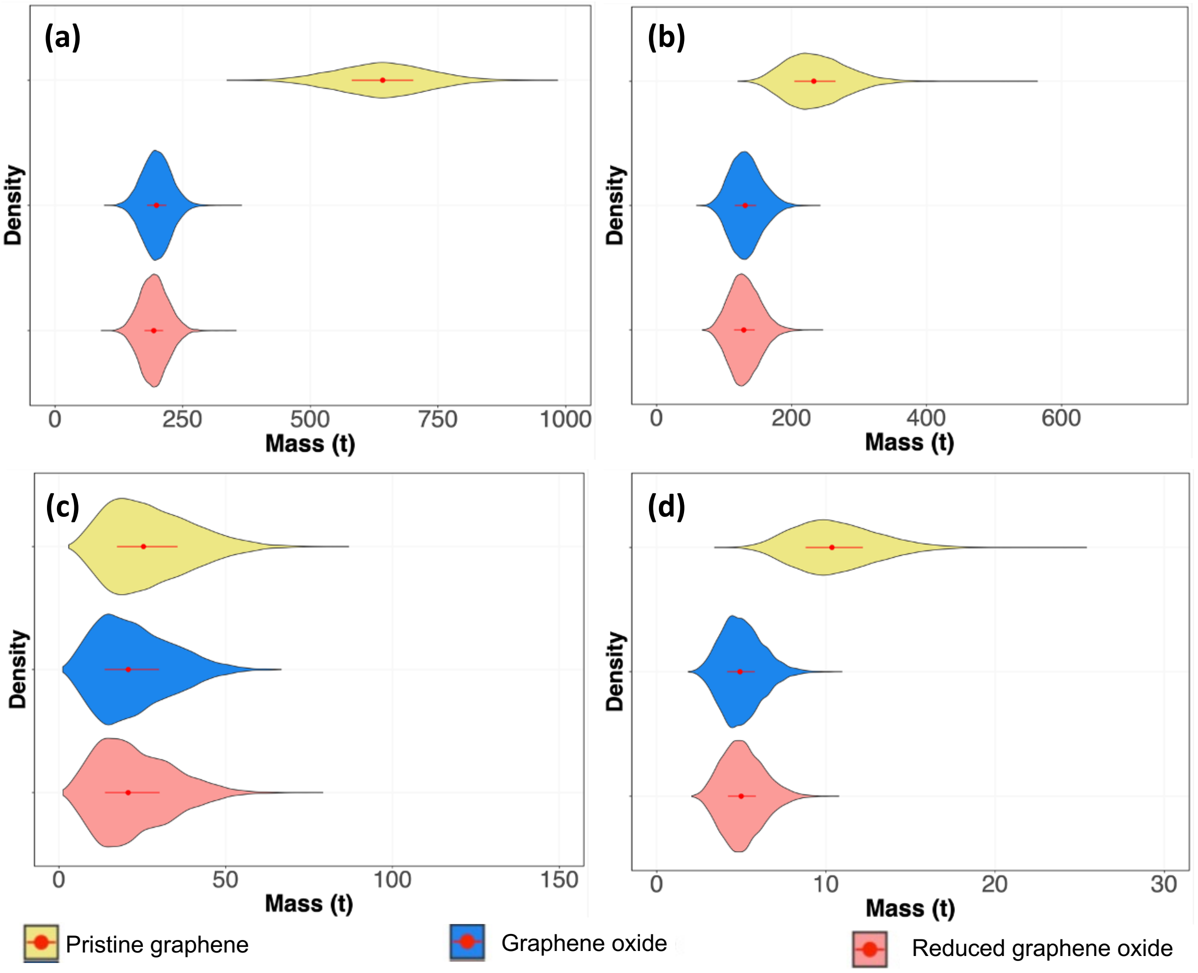


Figure S 3: Example of probability distributions of GBM accumulated masses in different sinks. Two-sided kernel density plot of all forms of GBM by 2030 for (a) *the elimination*, (b) *the landfill*, (c) *the subsurface* and (d) *the surface water* compartment. Shown are pristine graphene (yellow), graphene oxide (blue), and reduced graphene oxide (red). The red dots show the means and the red lines show the interquartile range of the distributions.

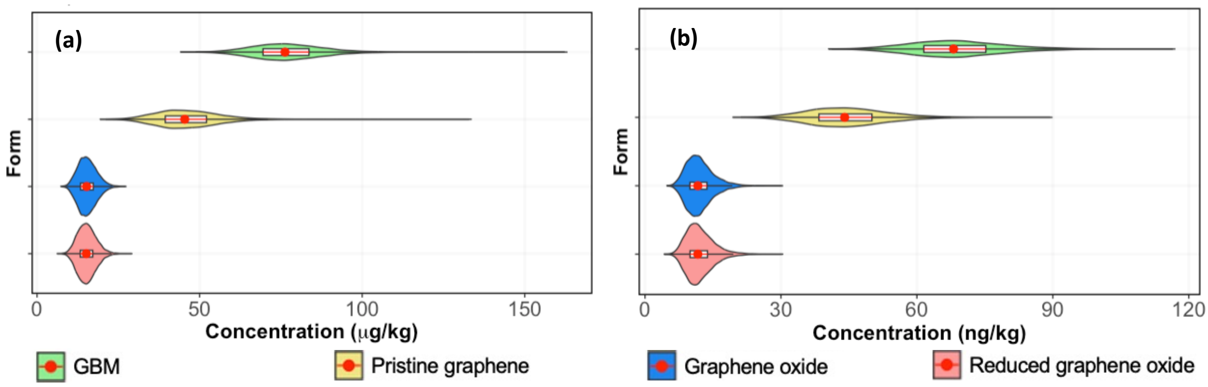


Figure S 4: Predicted environmental concentration of GBM and all forms of GBM in 2030 in (a) European sludge treated soil and (b) natural and urban soil. The red dots show the means and the red lines show the interquartile range of the distributions.

Table S 4: Summary of the Predicted Environmental Concentration distribution of pristine graphene (pG), graphene oxide (GO) and reduced graphene oxide (rGO) in 2030 (Median, Mean, 5th, 25th, 75th, and 95th Percentiles). NU soil: natural and urban soil; ST soil: sludge treated soil.

| | Form | Units | $Q_5$ | $Q_{25}$ | Median | Mean | $Q_{75}$ | $Q_{95}$ |
|---|---|---|---|---|---|---|---|---|
| NU soil | pG | ng/kg | 31.12 | 38.37 | 44.04 | 44.60 | 50.06 | 60.05 |
| | GO | ng/kg | 7.94 | 9.92 | 11.65 | 12.03 | 13.67 | 17.57 |
| | rGO | ng/kg | 7.97 | 9.94 | 11.65 | 12.08 | 13.78 | 17.64 |
| ST soil | pG | µg/kg | 32.01 | 39.44 | 45.43 | 46.16 | 52.11 | 62.57 |
| | GO | µg/kg | 11.06 | 13.39 | 15.24 | 15.43 | 17.29 | 20.44 |
| | rGO | µg/kg | 11.01 | 13.29 | 15.15 | 15.30 | 17.12 | 20.12 |
| Surface water | pG | ng/L | 0.37 | 0.52 | 0.67 | 0.71 | 0.85 | 1.18 |
| | GO | ng/L | 0.19 | 0.27 | 0.33 | 0.34 | 0.41 | 0.54 |
| | rGO | ng/L | 0.19 | 0.26 | 0.33 | 0.34 | 0.41 | 0.54 |

Table S 5: Summary of the Predicted No Effect Concentration (PNEC) distributions for the different forms of GBM (in micrograms per liter) (Mean, 5th, 25th, 75th, and 95th Percentiles). pG, GO and rGO indicates pristine graphene, graphene oxide and reduced graphene oxide, respectively.

| | $Q_5$ | $Q_{25}$ | Mean | $Q_{75}$ | $Q_{95}$ |
|---|---|---|---|---|---|
| GBM | 5 | 9 | 12 | 15 | 21 |
| pG | 2 | 13 | 22 | 31 | 41 |
| GO | 5 | 11 | 14 | 17 | 21 |
| $rGO_{AF}$ | - | - | 34 | - | - |
